# Construction and Validation of a UPR-Associated Gene Prognostic Model for Head and Neck Squamous Cell Carcinoma

**DOI:** 10.1155/2022/8677309

**Published:** 2022-06-06

**Authors:** Tao Wang, Lingling Chen, Fuping Xie, Shiqi Lin, Yuhan Lin, Jiamin Chen, Huanhuan Liu, Ye Wu

**Affiliations:** ^1^Fujian Key Laboratory of Oral Diseases and Fujian Provincial Engineering Research Center of Oral Biomaterial and Stomatological Key Lab of Fujian College and University, School and Hospital of Stomatology, Fujian Medical University, China; ^2^School of Stomatology, Fujian Medical University, China

## Abstract

Our study is aimed at constructing and validating a UPR-associated gene signature to predict HNSCC prognosis. We obtained 544 samples of RNA sequencing data and clinical characteristics from TCGA database and randomly grouped the samples into training and testing cohorts (1 : 1 ratio). After identifying 14 UPR-associated genes with LASSO and univariate Cox regression analysis, HNSCC samples were categorized into low-risk (LR) and high-risk (HR) subgroups depending on the risk score. Our analyses indicated that low-risk patients had a much better prognosis in the training and testing cohorts. To predict the HNSCC prognosis with the 14 UPR-associated gene signatures, we incorporated the UPR gene risk score, N stage, M stage, and age into a nomogram model. We further explored the sensitivity to anticancer drugs by using the IC50 analysis in two subgroups from the Cancer Genome Project database. The outcomes showed that the AKT inhibitor III and sorafenib were sensitive anticancer drugs in HR and LR patients, respectively. The immune cell infiltration analysis and GSEA provided strong evidence for elucidating the molecular mechanisms of UPR-associated genes affecting HNSCC. In conclusion, the UPR-associated gene risk score, N stage, M stage, and age can serve as a robust model for predicting prognosis and can improve decision-making at the individual patient level.

## 1. Introduction

Head and neck squamous cell carcinoma (HNSCC) is the seventh most common malignant neoplasm globally. There were an estimated 931.7 thousand new cases of HNSCC worldwide in 2020, and more than 467,100 individuals died of this disease [1–3]. Smoking, alcohol abuse, and areca nut chewing are risk factors for HNSCC. Recent studies have shown that viruses may be extremely associated with an increased risk of HNSCC development, together with persistent infections by EBV and HPV [4–7]. HNSCC is characterized by a high degree of malignancy, high metastasis rate, and poor clinical prognosis [8]. The 5-year survival rate of HNSCC is less than 50% [4, 6, 9]. The prognosis of HNSCC is connected directly to the tumor stage, cervical lymph node, and distant metastasis. The TNM staging system of the AJCC is widely used to evaluate the prognosis of HNSCC clinically. However, there is a common phenomenon in clinical practice: patients with similar clinical stages show different prognostic outcomes to some extent, which suggests that TNM staging is not an accurate predictor of survival. Therefore, it is urgent to find an effective and reliable biomarker to help doctors assess the prognosis of patients accurately and formulate a personalized diagnosis and treatment plans.

Activation of the unfolded protein response (UPR) plays an essential role in tumor transformation. The UPR is chronically activated in general tumor cells, and it is believed that this state is a mechanism that leads to antiapoptosis and drug resistance of tumor cells [10–14]. When cancer cells suffer from internal and external challenges such as oncogene activation and hypoxia, misfolded proteins accumulate in the endoplasmic reticulum (ER) lumen [15]. To maintain the ER in a stable environment under this condition, cells initiate signaling cascades that reduce protein synthesis, upregulate the expression of chaperones and folding enzymes, and induce accelerated degradation of misfolded proteins. Adaptive changes in cells make up the UPR [16].

Cells may adapt to adversity and survive by initiating autophagy via the UPR. However, the UPR transforms from a cellular protective response to a cytotoxic response that promotes apoptosis when ER stress is not alleviated [10]. Cancer cells hijack the UPR by activating UPR sensors, such as ATF6, IRE1a, and PERK, and their main regulatory factors, such as GRP78, allow drug resistance. Therefore, inhibiting the UPR pathway makes cancer cells more sensitive to conventional/targeted drug therapy [16, 17]. A recent study [18] found that tumor cells escaped the immune response by modulating immune cell activity in the tumor microenvironment via the UPR. Consequently, the UPR may provide a new way to judge the prognosis of patients with HNSCC. However, few investigations have explored whether the UPR is associated with survival outcomes in HNSCC.

Our study screened UPR-associated genes that were strongly connected with the prognosis of HNSCC. Fourteen UPR-associated genes were verified as prognostic biomarkers by survival analysis. The half-maximal inhibitory concentration (IC50) analysis, immune infiltration analysis, and a prognostic nomogram were carried out to help improve the understanding of survival prediction for HNSCC patients. Our study elucidated the molecular mechanism of UPR-associated genes affecting HNSCC, and UPR-associated gene may provide prognostic guidance for clinical diagnosis and treatment.

## 2. Materials and Methods

### 2.1. Data Acquisition of HNSCC Datasets

We downloaded and extracted the clinical and raw RNA-seq info of 500 HNSCC patient samples and 44 paracancerous samples from TCGA database. HNSCC patients were randomized to training and testing cohorts (1 : 1 ratio). The microarray expression and clinical data of a tongue cancer patient cohort (GSE41613) were used for external verification of signatures, which were obtained from the GEO database.

### 2.2. ssGSEA

The HNSCC RNA-seq expression data were analyzed by using the ssGSEA method with the GSVA [19] package according to the hallmark gene set. The univariate Cox regression was carried out according to ssGSEA values and HNSCC clinical information. The hallmark items were ultimately filtered for subsequent analysis according to the Cox results. Considering the criteria of *p* < 0.05 and maximum HR value, we selected UPR for subsequent analysis.

We performed WGCNA [20] with the WGCNA R package based on the HNSCC RNA-seq data of each gene and screened target genes. The correlation between the screened hallmark ssGSEA value and each coexpression module was calculated. The modular genes with the best correlation (the largest absolute value of the correlation coefficient) were selected for subsequent analysis.

The genes in the selected coexpression module had the best correlations with the ssGSEA value of the specified item. According to the clinical data, the survival [21] package was applied for the univariate Cox regression analysis on each gene, and genes with *p* < 0.01 were selected for LASSO model construction.

### 2.3. Construction and Validation of the UPR-Associated Gene Prognostic Signature

The glmnet [22] package was used to perform the LASSO regression analysis on the basis of the gene expression and clinical data acquired from the training group corresponding to the above screening results. After calculating the regression coefficients corresponding to each gene, the marker gene with regression coefficients not equal to 0 was determined. Survival and receiver operating characteristic (ROC) analyses were performed to verify the impact of the risk score on the prognosis of HNSCC patients and generate ROC curves based on the risk score and clinical information. According to the LASSO model and the expression level of each gene, the predict.cv.glmnet function was used to compute the risk score of each sample in the two groups.

Samples in the training and testing groups were divided into HR and LR groups according to the median risk score for survival analysis. ROC curve analysis was carried out to verify the LASSO regression model results. Combining the training and testing group data, we verified the LASSO regression model results and performed ROC analysis again. The GSE41613 dataset was used for external validation. The Cox regression model obtained from the training group was used to predict the risk of each sample in GSE41613, in which unrecorded genes were replaced by 0 values.

We grouped TCGA cohort by T stage, N stage, M stage, sex, age, histological grade, and pathological group. The Wilcox.test function in the R package was used to test the risk score in the above groups, and the correlation between some clinical data and the risk score was calculated.

### 2.4. Analysis of Sensitivity to Anticancer Drugs

According to the CGP database and the expression levels of UPR-associated genes in each HNSCC sample, the pRRophetic R package [23] was used for the IC50 test. After dividing the samples into HR and LR groups of UPR-associated gene expression by the median risk score, we calculated the IC50 score between the two groups with the limma [24] R package.

### 2.5. Analysis of Immune Infiltration

The CIBERSORT [25] software was used to score the immune infiltration of each sample according to the expression values of genes. The correlations between the scores of all 22 immune cells were estimated. The Wilcoxon test was applied to test the differences in immune scores between the HR and LR groups.

### 2.6. Construction and Verification of the Nomogram Model

We developed a nomogram using the rms package and combined the risk score with various clinical factors obtained in the LASSO analysis to perform a nomogram analysis. Afterward, the nomogram risk score of each sample was calculated. The survival, ROC, and Cox analyses were finally verified.

## 3. Results and Discussion

### 3.1. Construction of the UPR-Associated Gene-Bas Prognostic Signature

**Figure 1:**
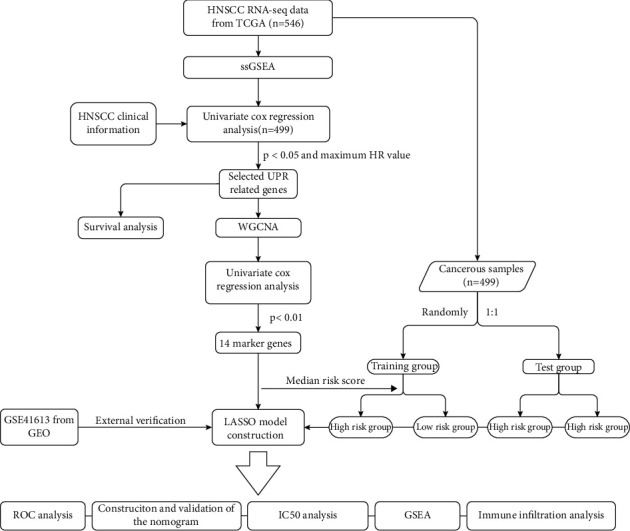
Flow diagram for developing and validating a UPR-associated gene prognostic model.

**Figure 2:**
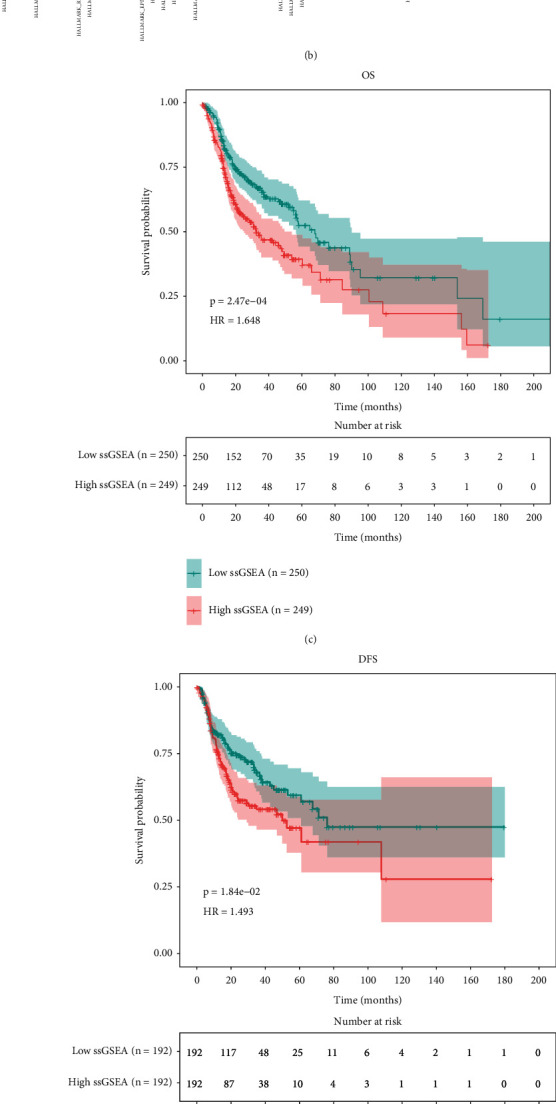
A prognostic signature based on genes associated with UPR. (a) Heatmap of the ssGSEA score of each item. (b) Heatmap of ssGSEA score correlations between items. (c) OS of the HR and LR groups. (d) DFS of the HR and LR groups. (e) Time-dependent ROC curves of the UPR-associated gene signature from 1 year to 10.5 years. (f) ROC curves for 3, 5, and 7 years.

The workflow of our investigation is demonstrated in Figure 1. First, we enrolled a total of 544 samples with RNA-seq data and clinical information, including 44 paracancerous samples from TCGA database. After ssGSEA with the GSVA package according to the hallmark gene set, the HNSCC cohort samples were subjected to the univariate Cox regression analysis to explore pathways associated with prognosis. A total of 499 cancerous samples with survival information were used in this procedure. Considering the criteria of *p* < 0.05 and maximum HR value, we selected UPR-associated genes for subsequent analysis (Figures 2(a) and 2(b) and Table 1). According to the median ssGSEA scores in the UPR gene set, the HNSCC cohort samples were divided into HR and LR groups, and survival analysis was performed. The overall survival (OS) and disease-free survival (DFS) of HNSCC were significantly different between the two groups (Figures 2(c) and 2(d)). ROC curves from 1 year to 10.5 years (Figures 2(e) and 2(f)) revealed that the areas under the curve (AUCs) were all greater than 0.5, and the best cutoff value was 0.607 (5 years). The results indicate that the UPR-associated gene signature may predict long-term survival in patients with HNSCC.

A coexpression analysis was applied using the WGCNA package, and the correlations between the ssGSEA score of the hallmark UPR-associated gene and each coexpression module were calculated. Genes in the module with the best correlations were selected for subsequent analysis. According to clinical data, the survival package was used for the Cox regression analysis of the selected genes, and candidate genes (*p* < 0.01) were used in the LASSO model construction.

**Figure 3:**
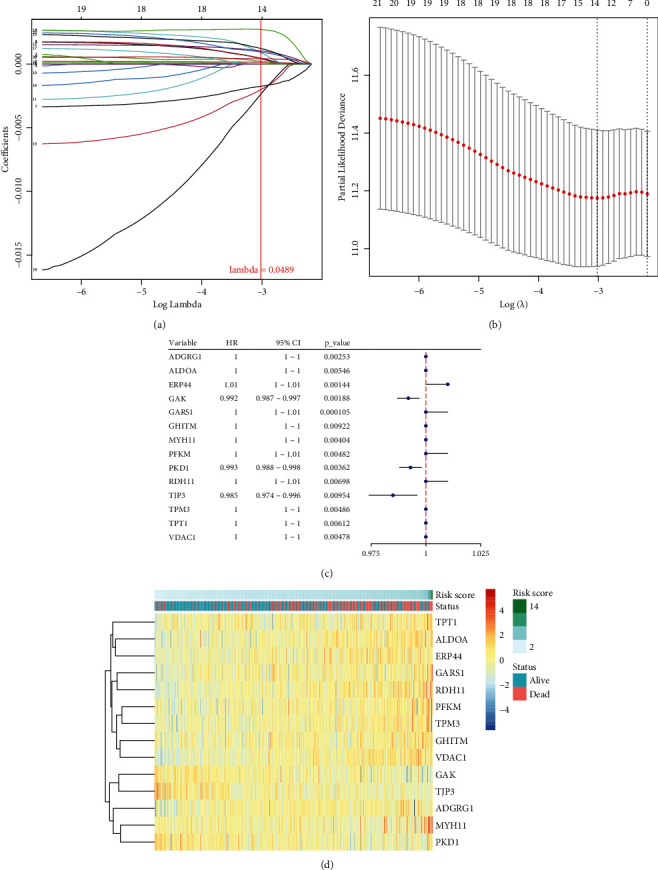
An analysis of the LASSO Cox and univariate Cox regression of the UPR gene signature. (a) LASSO regression results, *λ* = 0.0489. (b) Cross-validation diagram of LASSO regression results. (c) The univariate Cox regression diagram of marker genes. (d) Heatmap of marker genes, selected from the univariate Cox regression.

At a 1 : 1 ratio, 499 samples were randomly divided into test and training groups. Based on the expression of these candidate genes in the training group and clinical data in TCGA database, the LASSO regression analysis was conducted. Genes with regression coefficients that were not equal to 0 in the LASSO regression analysis (lambda = 0.0489, min = 0.0276) were selected as marker genes (Figures 3(a) and 3(b)), and we obtained 14 marker genes (Figure 3(c)). A risk score was calculated for each sample using the predict.cv.glmnet function based on the Cox regression model and the expression levels of each gene (Figure 3(d)).

### 3.2. Evaluation of the UPR-Associated Gene Prognostic Signature in the Internal and External Validation Cohorts

**Figure 4:**
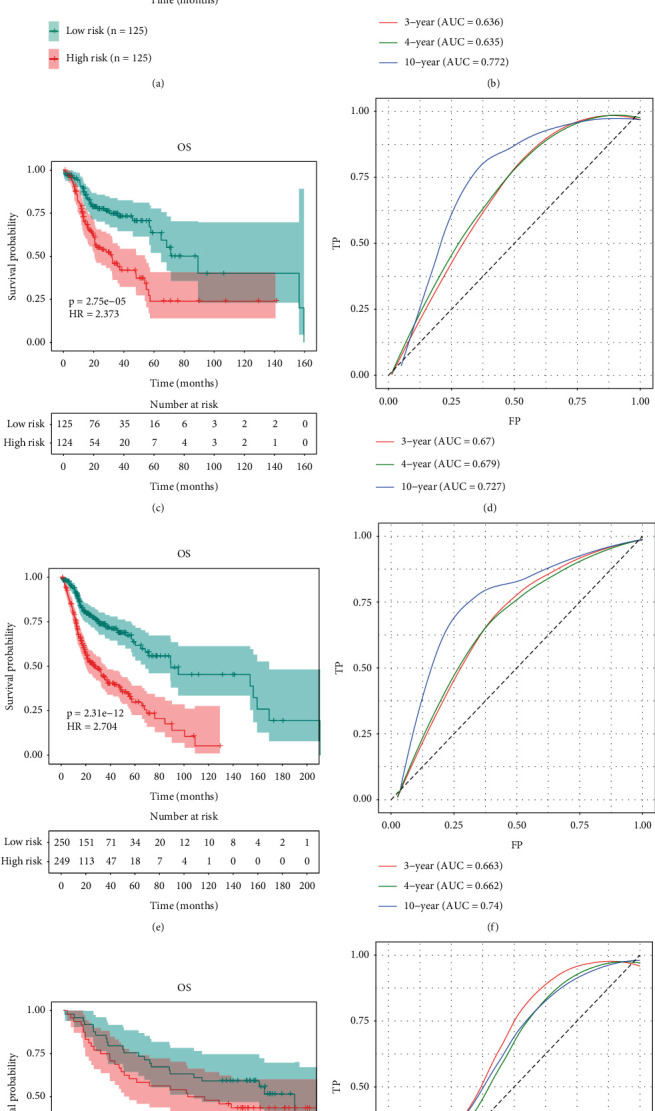
LASSO regression verification. (a, b) OS and ROC curves of the training group. (c, d) OS and ROC curves of the testing group. (e, f) OS and ROC curves of the total data of the training and testing groups. (g, h) OS and ROC curves of the external data (GSE41613).

Based on the median risk score, we divided the training and testing groups into HR and LR groups for the validation of the LASSO Cox regression results. The results demonstrated that the difference in OS was significant between the HR and LR groups (Figures 4(a) and 4(c)). The 10-year AUC of the training group was 0.772, and the 10-year AUC of the test group was 0.727 (Figures 4(b) and 4(d)). After merging the data, the results also showed the same trends for the HR and LR groups (*p* = 2.31*E* − 12, HR = 2.704), and the 10-year AUC was 0.74 (Figures 4(e) and 4(f)). Our results indicated that low-risk patients had a better prognosis in the training (HR vs. LR patients; 5-year OS: 8.8% vs. 14.4%; *p* < 0.001) and testing (HR vs. LR patients; 5-year OS: 5.6% vs. 12.8%; *p* < 0.001) cohorts.

External validation was performed with the GSE41613 dataset. The ROC analysis revealed a 5-year AUC of 0.607 (Figures 4(g) and 4(h)), and there was a significant difference in OS between the HR and LR groups (*p* = 3.33*E* − 01, HR = 1.312). LR patients also had a better prognosis (HR vs. LR patients; 5-year OS: 29.2% vs. 49.0%; *p* < 0.001). The results from the internal and external verification cohorts confirmed that UPR-associated genes affected HNSCC patient survival.

### 3.3. Identification of Biological Characteristics Associated with the UPR-Associated Gene Prognostic Signature

**Figure 5:**
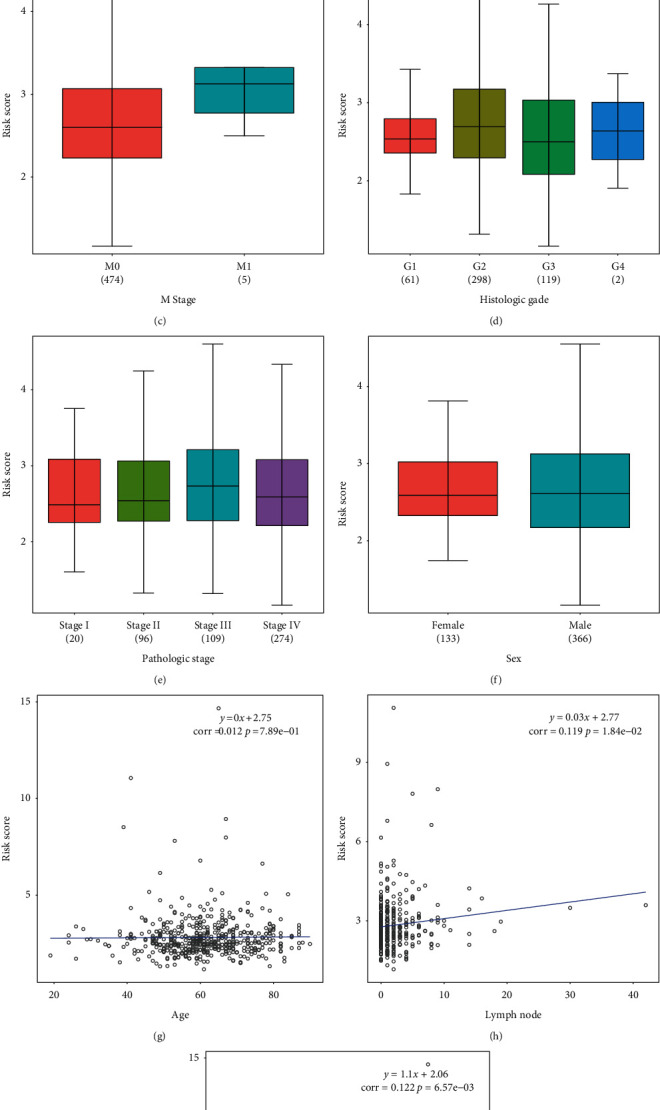
Clinical characteristics. Relationship between the risk score and clinical data in the (a) T stage, (b) N stage, (c) M stage, (d) histological grade, (e) pathological stage, and (f) sex groups. Correlation between the risk score and (g) age, (h) lymph node, and (i) tumor purity groups.

Further exploration of the relationship between UPR-associated genes and clinical features was performed by studying the differences in risk scores by histopathological grade, sex, age, T stage, N stage, and M stage. The difference in the risk score in each group is shown in Figures 5(a)–5(f). According to these results, sex, M stage, and pathological stage did not affect the risk score. In Figures 5(g)–5(i), tumor purity, lymph node status, and age were all factors that were related to the risk score.

### 3.4. Construction and Validation of the Nomogram

**Figure 6:**
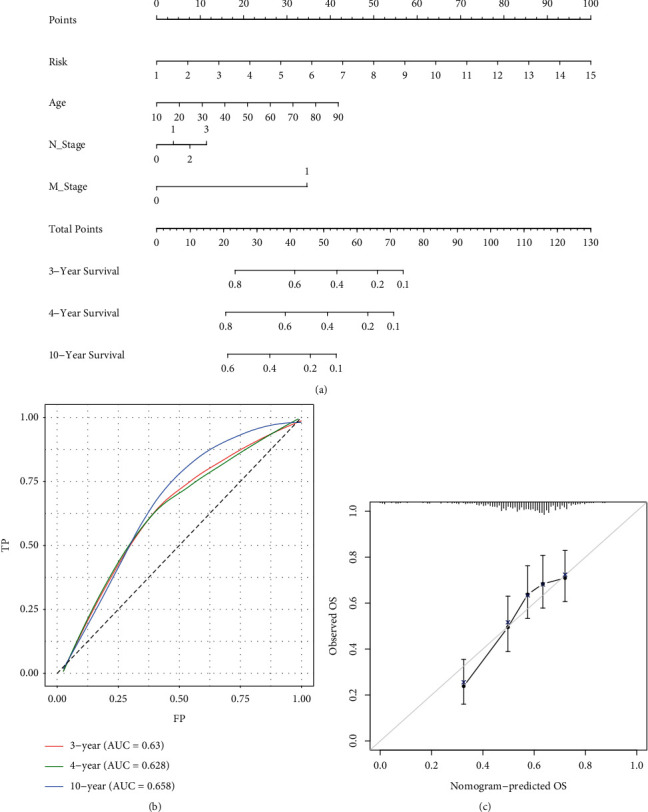
An algorithm based on the UPR-associated gene signature was constructed and validated. (a) Nomogram for predicting the OS probability of HNSCC patients. (b) Nomogram ROC curve analysis with time dependence. (c) The calibration of the nomogram for predicting OS.

Although multiple prognostic factors were selected, the complex interrelationships among variables and the contribution of each factor to tumor formation and development remain unclear. Therefore, a more comprehensive prognostic prediction model is needed. In this study, we created a nomogram model consisting of age, N stage, M stage, and the risk score based on the point scale to predict survival in HNSCC patients (Figure 6(a)). The nomogram model predictive accuracy was evaluated using calibration curves and ROC curves (Figures 6(b) and 6(c)). Based on our results, the nomogram model is able to accurately predict OS for HNSCC patients.

### 3.5. Anticancer Drug Responses

**Figure 7:**
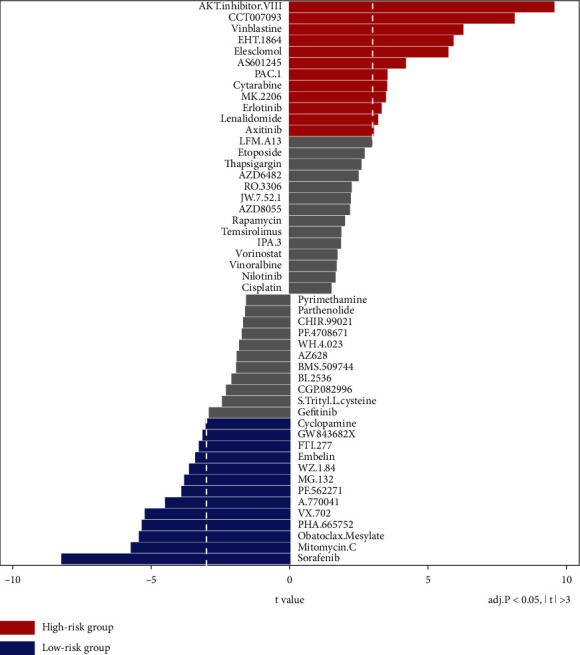
A half-maximal inhibitory concentration (IC50) analysis in the HR and LR groups.

To predict the response of a cancer patient to a therapeutic agent, we performed research on the difference in the IC50 score between the HR and LR UPR-associated gene groups. The outcomes (Figure 7) showed that anticancer drugs such as AKT inhibitor III, CCT007093, vinblastine, EHT 1864, elesclomol, and AS601245 were the most sensitive drugs in HR patients. Sorafenib, mitomycin C, obatoclax mesylate, PHA665752, and VX702 were the most sensitive anticancer drugs in the LR patients. Based on these findings, guidance for clinical treatment, which may vary depending on the type of UPR-associated gene, is provided.

### 3.6. Immune Infiltration Analysis

**Figure 8:**
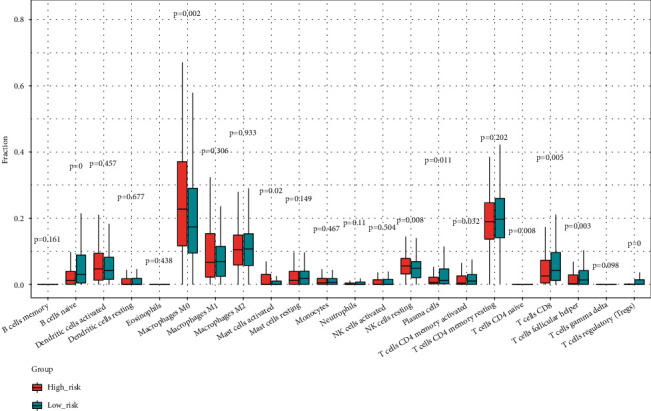
Immune cell infiltration analysis between the two test groups.

To explore the immune cells that may remarkably differ between different risk groups, we performed an immune infiltration analysis. The differences in M0 macrophages, follicular helper T cells, naive CD4 T cells, CD8 T cells, and resting NK cells were significant between the two test groups (Figure 8), which indicates that these immune cells may be associated with UPR-associated gene.

### 3.7. Gene Set Enrichment Analysis (GSEA)

**Figure 9:**
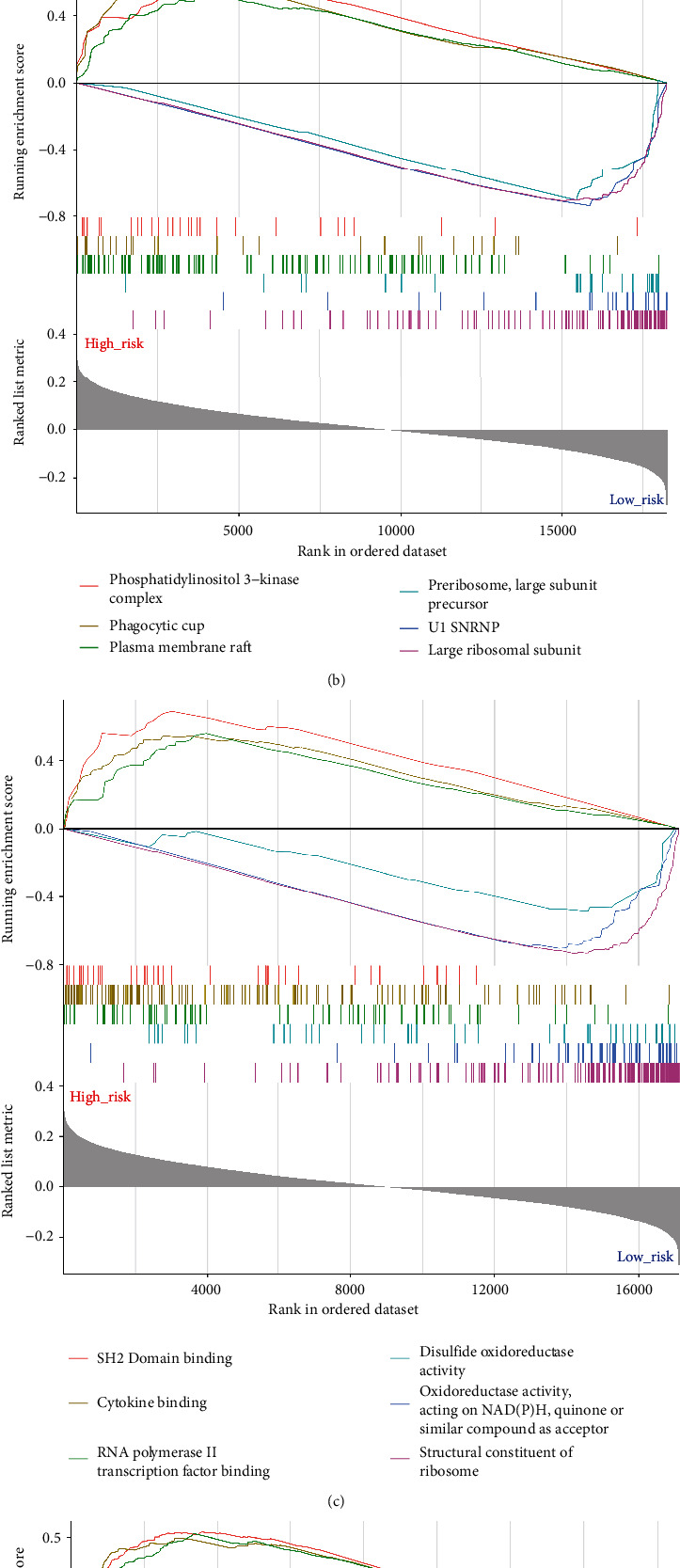
Gene set enrichment analysis (GSEA) in the HR and LR groups. The graph shows six items in (a) biological processes, (b) cellular components, (c) molecular functions, and (d) KEGG pathways.

We performed GSEA to understand the potential biological processes of UPR-associated gene, cellular components, molecular functions, and pathways that may vary considerably between different risk groups. As shown in Figure 9(a), the positive regulation of phosphatase activity, cellular extravasation, and positive regulation of phosphatidylinositol 3-kinase activity were significantly enriched in the HR group. The establishment of protein localization to the ER, protein targeting to the membrane, and protein localization to the ER was highly enriched in the LR group. Cellular component analysis showed that UPR-associated genes were related to the phosphatidylinositol 3-kinase complex, phagocytic cup, and plasma membrane raft in the HR group, and the preribosome large subunit precursor, U1 snRNP, and large ribosomal subunit were enriched in the LR group (Figure 9(b)).  Figure 9(c) shows that SH2 domain binding, cytokine binding, and RNA polymerase II transcription factor binding were highly enriched in the HR group, and disulfide oxidoreductase activity, which acts on NAPDH/quinone or similar compounds as acceptors, and structural constituents of ribosomes were enriched in the LR group. Platelet activation, gap junctions, and the thyroid hormone signaling pathway were related to UPR-associated genes in the high-risk group. Oxidative phosphorylation, olfactory transduction, and ribosomes were enriched in the LR group in the KEGG pathway analysis (Figure 9(d)). By analyzing enrichment analysis results, we were able to understand the molecular mechanisms underlying the UPR-associated genes affecting HNSCC, clarifying their role in affecting prognosis.

## 4. Discussion

The UPR is an adaptive signaling network that is evoked by physiological and pathological conditions. Researchers have examined the relationship between UPR activation markers and the prognosis of cancer [26, 27]. The results demonstrated that activation of the UPR was related to shorter OS, increased tumor aggressiveness, and increased metastasis in breast cancer, colorectal carcinoma, glioblastoma, and hepatocellular carcinoma [28–30]. Accumulated evidence also suggests that the UPR signaling pathway and ER stress play a functional role by regulating crucial tumor biological processes in HNSCC, including progression and therapy resistance [16]. However, few studies have revealed whether UPR status predicts prognosis in HNSCC patients. Additionally, HR patients with different UPR statuses should also be assessed for their immune status to increase the effectiveness of their immunotherapy. Therefore, our study is aimed at constructing a model based on UPR-associated genes for predicting HNSCC prognosis and further characterizing the IC50 scores and immune infiltration levels between the two UPR risk groups. The most significant conclusion from this study is that UPR status is an important determinant of prognosis in HNSCC patients, and the UPR-associated gene risk score combined with age, N stage, and M stage may be used to develop a robust prediction model for survival analysis. The IC50 analysis and immune infiltration analysis may improve decision-making at the individual patient level.

The univariate Cox analysis identified 14 key UPR-associated genes that affected HNSCC prognosis (ADGRG1, ALDOA, ERP44, GAK, GARS1, GHITM, MYH11, PFKM, PKD1, RDH11, TJP3, TPM3, TPT1, and VDAC1). ER-resident protein 44 (ERP44) is a redox sensor and regulates the location of critical enzymes that operate in the ER. ERP44 promotes progression in nasopharyngeal carcinoma via its interaction with ATP citrate lyase and regulation of epithelial-mesenchymal transition (EMT) [31]. Nasopharyngeal cancer cells release exosomes expressing ERP44, which may be delivered to adjacent cells to enhance chemoresistance under ER stress [32]. The above information suggests that ERP44 is an important gene influencing tumor progression and chemoresistance in HNSCC. Protein kinase D1 (PKD1) is a member of the serine/threonine kinase family and activates protective signals against ER stress. Several investigations have shown that PKD1 plays a role in the regulation of various tumor-related pathways [33]. PKD1 is closely related to the redifferentiation of keratinocytes and the increase in cell proliferation, and it may enhance the activity of the ERK/MAPK pathway [34]. Higher expression of PKD1 correlated with poor differentiation in oral squamous cell carcinoma [35]. PKD1 is frequently downregulated at both the transcriptional and protein levels in HNSCC cell lines [36]. However, there are no related reports about the effects of PKD1 on the prognosis of HNSCC. MYH11 is a novel gene for predicting OS in HNSCC and may be a drug target based on bioinformatic analysis [37, 38], but experiments supporting these findings have not been conducted. The effects of PFKM, ADGRG1, ALDOA, GAK, RDH11, TJP3, TPM3, TPT1, and VDAC1 on the prognosis of HNSCC and the immunological changes in the HNSCC microenvironment have rarely been reported and need further investigation. We also demonstrated that the UPR-associated genes were differentially expressed in different T stage, N stage, and histological grade groups. We revealed that the UPR risk score was related to cancer purity, lymph node status, and age. These results indicated that UPR-associated genes affected the biological behavior of HNSCC.

Several differences emerged between the prediction results of the model and the external validation results of the GSE41613 dataset. We speculate that this result may be related to the classification of HNSCC. HNSCC is divided into oral squamous cell carcinoma (OSCC), oropharyngeal carcinoma, nasopharyngeal carcinoma, laryngeal carcinoma, etc. The pathogenic factors of these cancers are diverse, and survival outcomes differ in the different subsites of HNSCC [39]. Therefore, HNSCC information in TCGA database is relatively mixed. Deviations in the grouping of the training cohort and test cohort may lead to the above unsatisfactory results. Therefore, we suggest that future analysis of the prognosis at one subsite of HNSCC, such as OSCC, should be based on the data of this subsite rather than on HNSCC with mixed multiple sites. However, this model has the principal benefit of bringing a complementary perspective to individual tumors and establishing a scoring framework for patients. Therefore, our nomogram may be a favorable tool for clinicians in the future. However, whether the UPR-associated gene model predicts the recurrence of HNSCC is not known. This relationship is our future focus, and we will investigate the role of UPR-associated genes in the recurrence of HNSCC.

It is becoming increasingly recognized that heterogeneity is significant in tumor progression and clinical decisions. The present evidence supports that chemotherapy resistance in cancers is linked to the UPR and ER stress [32]. One problem with the existing anticancer drugs is that a particular drug shows different sensitivities in different individuals. Targeting UPR branches is a promising way to enhance the efficacy of chemotherapy for cancers [40]. Considering the above problem, we performed a UPR-related IC50 analysis to explore sensitivities and help HNSCC patients obtain personalized medication regimens. AKT inhibitor III, CCT007093, vinblastine, EHT 1864, elesclomol, AS601245, etc. may be the most sensitive anticancer drugs in HR patients whose outcome survival is much poorer than that of LR patients. However, more research is needed to verify whether these drugs can achieve the predicted sensitivity. A recent study [41] explored the best metric for predicting drug sensitivity, and they found that the area above the dose-response curve was better than the IC50. To further verify the anticancer sensitivity of these drugs in different UPR risk groups, in vivo and in vitro experiments should be conducted.

Previous studies have shown that the UPR evades the immune response by regulating the tumor microenvironment. The ER stress state of tumor cells is transmitted to macrophages and dendritic cells (DC) in the microenvironment. This communication leads to the upregulation of the expression of some proinflammatory cytokines and chemokines, which inhibits the maturation of DCs. It also inhibits the activation of CD8+ T cells and secretes arginine by reducing the process of antigen presentation, which inhibits T cell activation and leads to tumor immune escape [18]. Therefore, we further investigated the role of the UPR in the tumor microenvironment by evaluating immune cell infiltration. The results revealed significant differences in M0 macrophages, follicular helper T cells, naive CD4 T cells, CD8 T cells, and resting NK cells between the HR and LR groups. This finding provides important insights into the tumor immune microenvironment affecting the UPR.

Different risk groups showed enrichment in different pathways in the GSEA. The UPR genes included in the signature were primarily involved in platelet activation, gap junctions, and the thyroid hormone signaling pathway in the HR group. The platelets seem to play a critical role in malignant tumor metastasis. Cancer metastasis is promoted by the interaction between platelets and circulating tumor cells (CTCs). CTCs activate platelets, and activated platelets accumulate and protect CTCs from NK cells and shear stress. Finally, CTC hypoxia tolerance is promoted by platelets, along with angiogenesis, EMT, extravasation, and ultimately metastasis [42]. Our results suggested an explanation for why HR group patients have a poorer outcome than LR patients, and the status of platelet activation may be a vital factor.

In summary, we identified 14 genes associated with the UPR in HNSCC patients that affected their prognosis. Based on these genes, we investigated the prognostic significance of the UPR risk score for HNSCC patients and established a nomogram prediction model combining this risk score with age, N stage, and M stage. The infiltration of immune cells in the microenvironment was further analyzed, which provided some information for immunotherapy in different risk groups. This study also described therapeutic regimens in different risk groups of HNSCC, and it may be used as a reference for further studies on clinical medication. Notably, the prognosis of HNSCC was analyzed from the perspective of the UPR, and the changes in the immune microenvironment and possible effective drug regimens were described, which provided certain help for the treatment of HNSCC. However, because our study was based on bioinformatic analysis, there are some limitations, and our results must be confirmed in further clinical studies. This limitation means that the study findings must be interpreted cautiously. The function and mechanism of these UPR-associated genes, either individually or in combination, should be investigated to support their clinical application.

## 5. Conclusions

We developed a potent model based on the UPR-associated gene signature, i.e., the UPR risk score combined with age, N stage, and M stage, and this model may be used to predict HNSCC survival prognosis. Our study enhances the understanding of genes associated with UPR pathways in HNSCC and can improve decision-making at the individual patient level.

## Figures and Tables

**Table 1:** Cox regression results of hallmark items.

Term	*p* value	HR
HALLMARK_MYC_TARGETS_V1	0.011703	179594.1
HALLMARK_MTORC1_SIGNALING	4.29*E* − 05	1.59*E* + 10
HALLMARK_OXIDATIVE_PHOSPHORYLATION	0.030742	1266.05
HALLMARK_PROTEIN_SECRETION	0.018633	141007.4
HALLMARK_TGF_BETA_SIGNALING	0.067779	381.6219
HALLMARK_UNFOLDED_PROTEIN_RESPONSE	3.63*E* − 05	5.51*E* + 12
HALLMARK_MITOTIC_SPINDLE	0.736746	0.291637
HALLMARK_MYC_TARGETS_V2	0.05255	122.3036
HALLMARK_G2M_CHECKPOINT	0.495757	5.585005
HALLMARK_REACTIVE_OXYGEN_SPECIES_PATHWAY	0.061818	2061.022

## Data Availability

The data presented in this study are openly available in TCGA and GEO databases.
